# Asymmetric organocatalytic synthesis of chiral homoallylic amines

**DOI:** 10.3762/bjoc.20.201

**Published:** 2024-09-16

**Authors:** Nikolay S Kondratyev, Andrei V Malkov

**Affiliations:** 1 Department of Chemistry, Loughborough University, Loughborough, LE11 3TU, UK; 2 Faculty of Science and Engineering, University of Wolverhampton, Wolverhampton, WV1 1LY, UK

**Keywords:** asymmetric catalysis, asymmetric synthesis, chiral amines, organicatalysis, rearrangement

## Abstract

In recent decades, the chiral allylation of imines emerged as a key methodology in the synthesis of alkaloids and natural products with 4-, 5- and 6-membered cyclic amine motifs. Initially reliant on stoichiometric reagents, synthetic chemists predominantly used *N*-substituted chiral imines, organometallic chiral reagents and achiral reagents with an equimolar chiral controller. However, recent years have witnessed the rise of asymmetric transition-metal catalysts and, importantly, organocatalytic allylation, reshaping the landscape of modern synthetic chemistry. This review explores the latest developments in the asymmetric allylation of imines, encompassing cutting-edge advances in hydrogen-bond catalysis and non-classical approaches. Furthermore, practical examples showcasing the application of these innovative methodologies in total synthesis are presented.

## Introduction

**Scheme 1:**
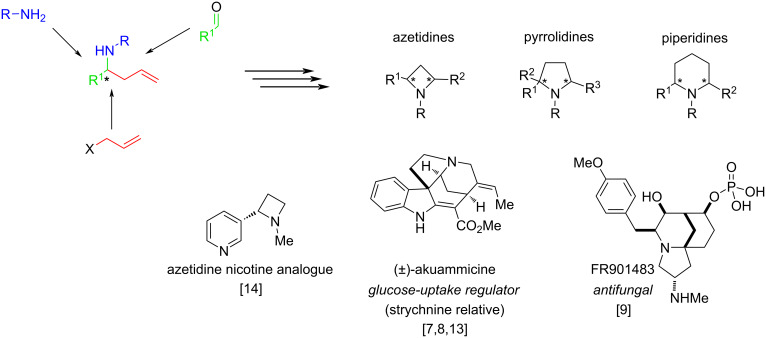
The position of homoallylic amines in the landscape of alkaloid and nitrogen compounds syntheses.

Nitrogen-containing organic compounds (sometimes referred to as alkaloids due to their basic properties) are of critical importance in medicinal chemistry because of their unique binding properties to biomolecules [1]. Out of 55 drug candidates, approved by the FDA in 2023, 28 (51%) are nitrogen-containing organic compounds, with many featuring amino groups adjacent to a stereogenic centre. Some reports suggest that 75% of all FDA-approved compounds are alkaloids [2]. The industrial production of amines on a million-ton scale underscores the significance of methods for their synthetic utilisation [3]. Both, nature and humanity utilise ammonia as the central precursor to introduce nitrogen into organic compounds. Nature achieves this through enzymatic processes, sourcing ammonia from geological origins or directly from the air [4], while humanity relies on the Haber–Bosch process and the rich toolkit of advanced organic synthesis [5]. Homoallylic amines occupy a significant niche in alkaloid synthesis as they frequently appear as key intermediates in syntheses of the various nitrogen-containing natural products [6–14]. Additionally, they can be directly prepared by combining three readily accessible synthons: an amine, an allyl nucleophile, and a carbonyl compound (Scheme 1) [15–16]. A selection of structural motifs accessible via homoallylic amines is shown in Scheme 1.

Despite several reviews on homoallylic amine syntheses being published [17–23], none have focused specifically on organocatalytic approaches, which are particularly important for medicinal chemistry due to their greener credentials. Given the wide range of organocatalytic methods for synthesising homoallylic amines developed in the past decade, it is essential to provide a comprehensive overview of this significant topic.

## Review

### Asymmetric allylation with boron-based reagents

**Scheme 2:**
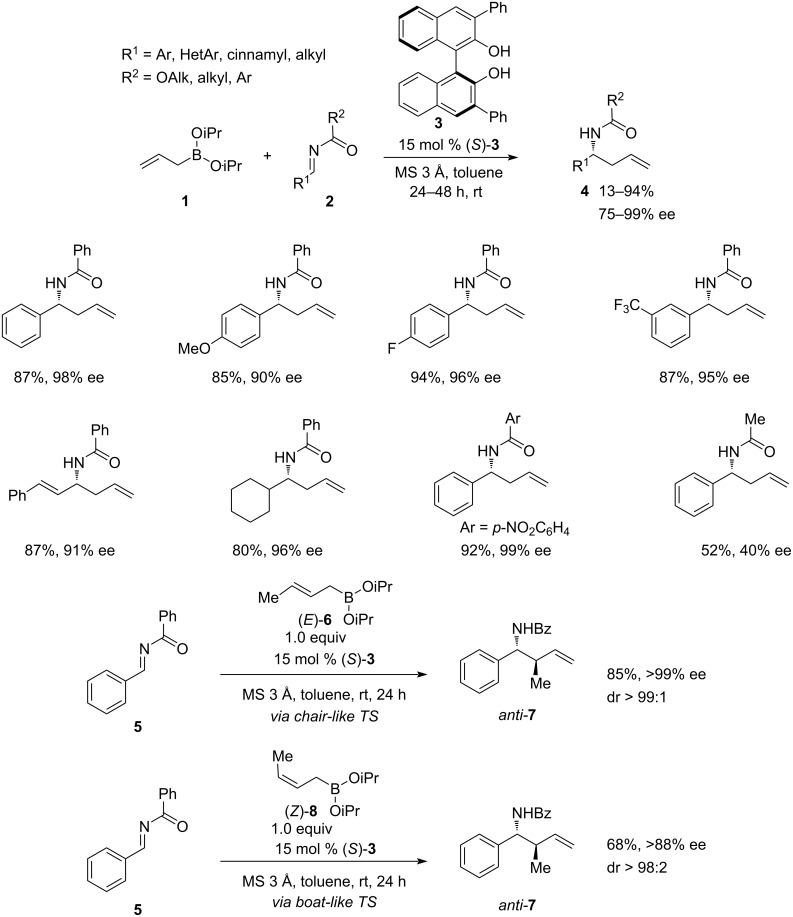
3,3’-Diaryl-BINOL-catalysed asymmetric organocatalytic allylation of acylimines [24].

The research on the metal-free, asymmetric organocatalytic allylation of acylimines was pioneered in 2007 by Schaus and co-workers [24]. In their elegant approach, high enantioselectivities (90–99% ee) and good yields (75–94%) have been achieved on a wide range of aromatic and aliphatic *N*-acylimines **2** using chiral 3,3’-diaryl-BINOL **3** as catalyst (Scheme 2).

The reaction proved to be highly tolerant to the nature of the R^1^ substituent in imine **2**, demonstrating high yields and enantioselectivities for both electron-rich and electron-poor *N*-benzoylarylimines, aliphatic *N*-benzoylimines, and bulkier *N*-carboxyalkylimines. However, less sterically hindered *N*-carboxyalkyl- and *N*-carbamoylimines showed a significant drop in both enantioselectivity and yield.

The observed enantioselectivity was attributed to the chair-like transition state where the organoboron reagent **1** exchanges one isopropoxy group for one of the BINOL **3** oxygen atoms, whereas the free OH group of the BINOL forms a hydrogen bond to the carbonyl of substrate **2**. The flanking phenyl groups on the BINOL facilitate recognition between two enantiotopic faces of acylimine **2**, exposing only one face to the attack by the allyl group. The replacement of one isopropoxy (iPrO) group between allylboronate **1** with BINOL was confirmed by ESI-MS and NMR analysis of the reaction mixture. Interestingly, both (*E*)- and (*Z*)-crotyl boronates **6** and **8** almost exclusively produced *anti*-homoallylamine **7**, with (*E*)-**6** being slightly more efficient. This was explained by the reaction switching from the chair-like TS in (*E*)-crotylation to the boat-like TS in (*Z*)-crotylation. The developed methodology was successfully applied for the total synthesis of Maraviroc, an HIV-1 drug of the CCR5-receptor antagonists class that was approved by the FDA in 2007.

**Scheme 3:**
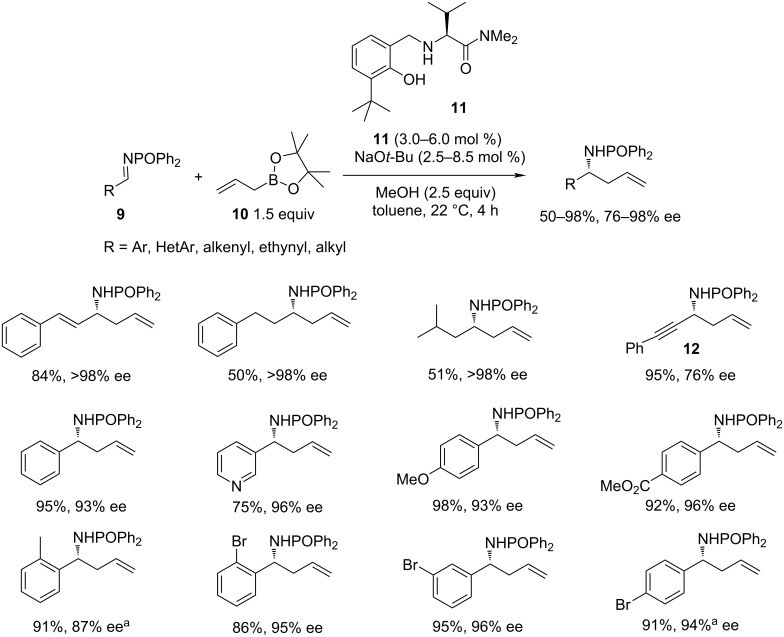
Aminophenol-catalysed reaction between *N*-phosphinoylimines and pinacol allylboronic ester. Imine scope [6]. ^a^Reaction time: 6 h.

In 2013, a significant work was published by Hoveyda and co-workers [6], who demonstrated that small organic molecules like hindered aminophenol **11** can serve as highly efficient and versatile catalysts in the asymmetric addition of organoboron reagents to imines (Scheme 3). An intermolecular hydrogen bonding between a non-rigid organocatalyst and a non-rigid substrate was shown to play a key role in assembling a configurationally stable transition structure. As a result, this approach unveiled the highly enantioselective nucleophilic addition of primary (**10**), secondary, and even tertiary allylboronates, as well as allenylboronates to a broad set of imines, bearing the *N*-phosphinoyl group. The new approach allowed the activation of both the substrate and the reagent using aminophenol organocatalyst **11**. It was proposed, that the internal hydrogen bond between the catalyst **11** and the P=O fragment of the protecting group of imine **9** is responsible for the observed high enantioselectivities (76–98% ee). The scope included a wide range of substrates, such as aromatic, heteroaromatic, aliphatic, and α,β-unsaturated imines **9**.

However, with ethynylimines **12**, the enantioselectivity dropped to a modest level. In addition, the reaction with sterically hindered tertiary allylboronates, such as the chiral (*R*)-α-cyclohexyl-α-methyl-allylboronate required the use of catalytic amounts of zinc *tert*-butoxide as an activator.

**Scheme 4:**
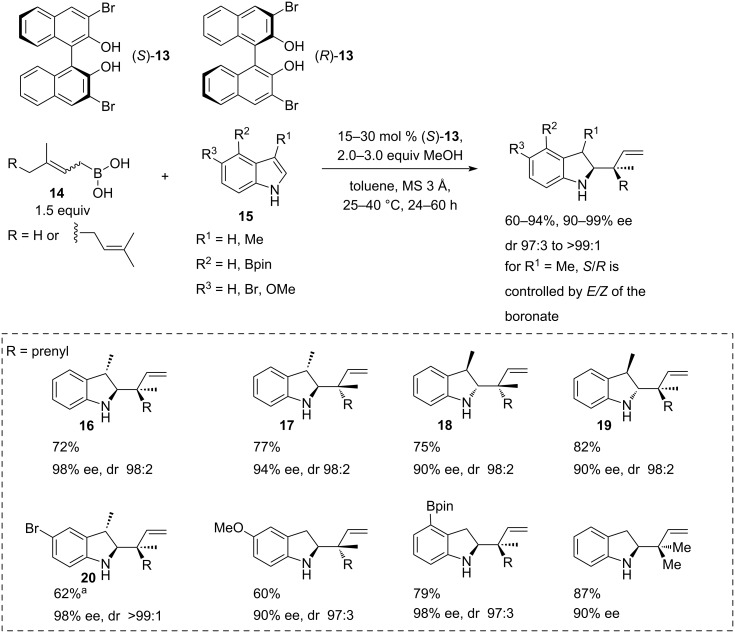
Asymmetric geranylation and prenylation of indoles catalysed by (*R*)- or (*S*)-3,3’-dibromo-BINOL [25]. ^a^Absolute configuration was confirmed by a single crystal XRD of the corresponding hydrochloride salt.

**Scheme 5:**
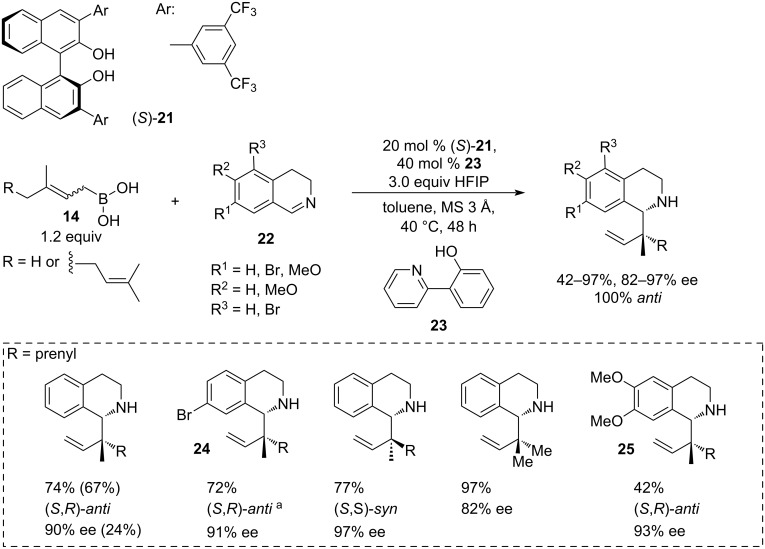
(*R*)-3,3’-Di(3,5-di(trifluoromethyl)phenyl-BINOL-catalysed asymmetric geranylation and prenylation of dihydroisoquinolines [25]. ^a^Absolute configuration was established by single crystal XRD of the corresponding hydrochloride salt.

Interesting examples of a direct asymmetric allylation of indoles **15** (Scheme 4) and 3,4-dihydroisoquinolines **22** (Scheme 5) with geranyl- and prenylboronic acids **14** in the presence of BINOL derivatives were reported by Szabó [25]. In the case of 3-methylindole, the methodology enabled simultaneous construction of homoallylamine scaffolds, exemplified by compounds **16**–**20** with up to 3 contiguous stereocentres, including a quaternary centre in moderate to high yields (60–94%) with 90–99% ee and dr in the range from 97:3 to >99:1 (Scheme 4).

The selectivity of the reaction arises from the reversible coordination of BINOLs **13** or **21** to the prenyl- or geranylboronic acids **14**, favouring one of the four possible chair-like transition states.

The reaction scope for indoles **15** was demonstrated on a limited set of 5-methoxyindole, 5-bromoindole, and 4-indolyl-pinacol boronates reacting with *E*- and Z-geranyl and prenylboronic acids **14** with yields in the range of 60–94%. *Z*-Geranylboronic acid (**14**, R = prenyl) proved to be less reactive, while stereoselectivities remained high (Scheme 4).

A modified procedure was developed for 3,4-dihydroisoquinolines **22**, which necessitated the use of 40 mol % of 2-(2-pyridyl)phenol (**23**) as an activator, 3 equivalents of HFIP and a slightly different catalyst, 3,3’-bis(3,5-bis(trifluoromethyl)phenyl)-BINOL **21** at 20 mol % loading (Scheme 5).

Interestingly, the reaction showed an opposite trend and worked better with *Z*-geranylboronic acid (**14**). The scope was tested over a few dihydroisoquinolines, including 7-bromo-3,4-dihydroisoquinoline and 6,7-dimethoxy-3,4-dihydroisoquinoline leading to **24** and **25**, respectively. The yields varied between 42–97% and were lower for the electron-rich substrates and higher for the electron-deficient substrates. The enantioselectivities were in the range of 82–97% and were lowest for the prenylation, and highest for *Z*-geranylboronic acid. The absolute configurations of the products were established by X-ray crystallography of the hydrochloride salts of the corresponding bromoindole and bromodihydroisoquinoline derivatives **20** and **24**, which supported the hypotheses regarding the selectivity-determining transition states arrangement.

It is important to note, that boronic acids **14** are highly sensitive to oxidation by air and could only be purified in air-free conditions and stored in airtight containers. Additionally, prenylboronic acid (R = H) was synthesised in only 31% yield. Given the 1.5 equiv loading of the boronic acids in the allylation protocol, the overall atom economy is not ideal.

**Scheme 6:**
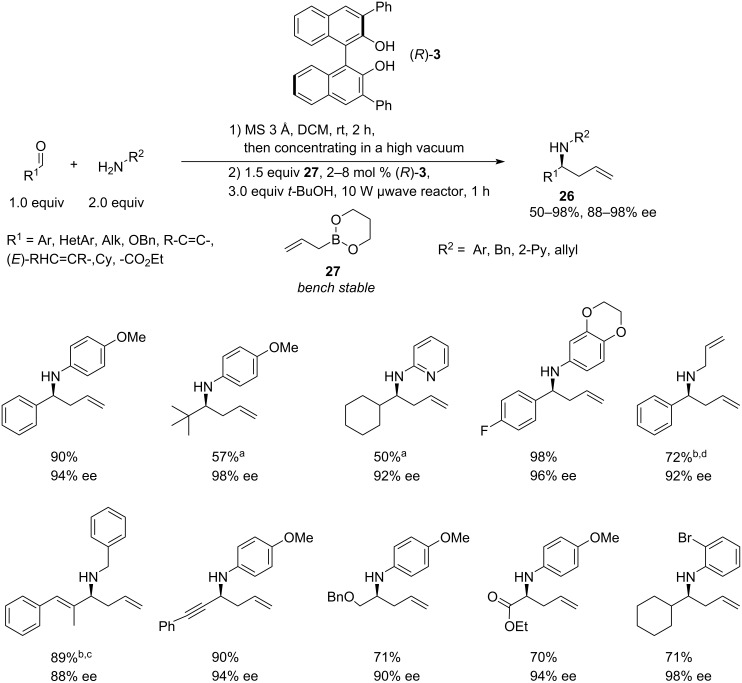
Microwave-induced one-pot asymmetric allylation of in situ*-*formed arylimines, catalysed by (*R*)-3,3’-diphenyl-BINOL. Aldehyde and amine scope [26]. ^a^The reaction was conventionally heated at 50 °C for 24 hours instead of microwave irradiation. ^b^1.0 equiv amine was used. ^c^8 mol % BINOL catalyst was used. ^d^4 mol % BINOL catalyst was used.

Asymmetric allylation of in situ-formed imines catalysed by chiral BINOLs received further development in the work of Schaus [26]. The new methodology used bench-stable allyl-1,3,2-dioxaborinane (**27**) in the reaction with preformed crude *N*-aryl-, *N*-benzyl- and *N*-allylimines in the presence of 2–8 mol % of the relatively simple 3,3’-Ph_2_-BINOL catalyst **3** at 50 °C in a microwave reactor at 10 W irradiation for 1 hour to afford amines **26** (Scheme 6).

**Scheme 7:**
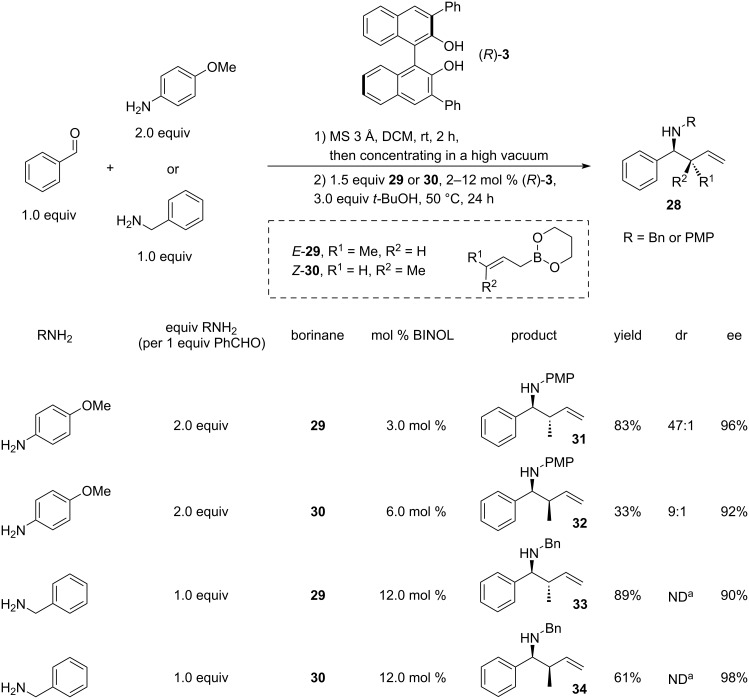
Microwave-induced one-pot asymmetric allylation of in situ*-*formed arylimines, catalysed by (*R*)-3,3’-diphenyl-BINOL. Crotylation studies [26]. ^a^Not determined, but estimated to be high; racemate did not produce 4 clear separate peaks on chiral HPLC.

The study explored a broad range of *p*-methoxyphenyl (PMP)imines derived from aryl, heteroaryl and alkyl aldehydes (including ethyl glyoxylate), demonstrating yields of 57–98% and high enantioselectivity of 90–98%. Screening of aldehyde and amine components indicated that the method is generally tolerant to diverse *N*-aryl-, *N*-heteroaryl-, and *N*-benzylimines. Additionally, the authors conducted asymmetric crotylations of *N*-(PMP)- and *N*-benzylimines using *E*- and *Z*-crotyldioxaborinanes **29** and **30** to furnish amines **28** (Scheme 7). With (*E*)-crotyldioxaborinane **29**, the *anti*-products **31** and **33** were achieved exclusively (dr > 20:1) in good yields (83% and 89%) and enantioselectivity (96% and 90%). On the other hand, the reactions with (*Z*)-crotyldioxaborinane **30** resulted in lower yields (33% for **32** and 61% for **34**). However, the diastereoselectivity towards the *syn* products remained notably high (9:1 and >20:1), along with impressive enantioselectivity levels (92% and 98% ee). A slight loss of diastereoselectivity in the reaction of the PMP-imine with (*Z*)-crotyldioxaborinane **30** was attributed to the spontaneous isomerisation of the imine to the *cis*-isomer. The crotylboronates were synthesised from the respective *cis* and *trans*-butenes by deprotonation followed by reaction with triisopropylborate [27], therefore the presence of *E* isomer in the *Z*-product **30** cannot be excluded.

Chiral BINOL-derived phosphoric acids have been known since the 1970s as industrially relevant chiral counterions for the resolution of chiral amines [28]. However, it was not until 2004 that they were recognised as efficient chiral Brønsted acid organocatalysts for asymmetric Mannich reactions [29].

**Scheme 8:**
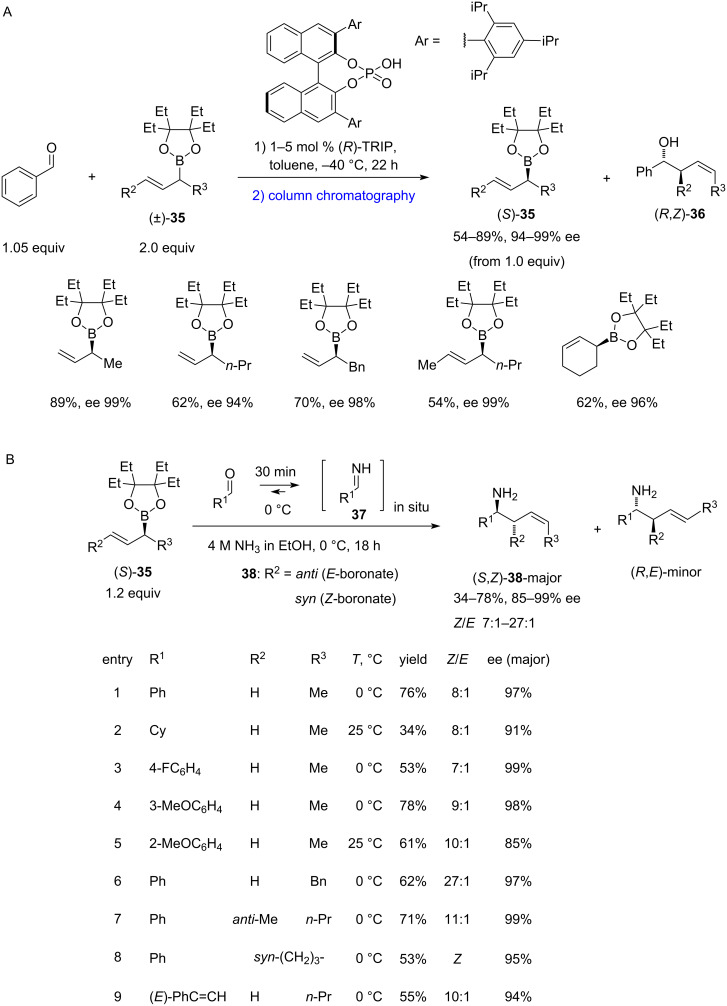
Kinetic resolution of chiral secondary allylboronates [15,30].

Malkov and co-workers revealed [30] that (*R*)-TRIP can act as a very efficient catalyst for the kinetic resolution of racemic, configurationally stable *sec*-allylboronates (±)-**35** (Scheme 8A). Using 2.5 equivalents of racemic boronate **35**, the reaction with aldehydes produced enantioenriched *Z*-homoallylic alcohols **36**. While this protocol was effective for aldehydes, it proved challenging to perform it with *N*-substituted aldimines.

This was later addressed by the same researchers [15], who developed an organocatalytic kinetic resolution of secondary boronates to furnish bench-stable homochiral (*S*)-**35**. The latter were then employed in the asymmetric allylation of in situ*-*formed primary aldimines **37**, leading directly to the unprotected chiral (*S*,*Z*)-homoallylic amines **38** in high yields and with an excellent retention of the enantiopurity (Scheme 8B). The method remains the only direct *Z*-selective crotylation to attain enantioenriched unprotected homoallylic amines, and the only example of an effective kinetic resolution of chiral secondary allylboronates **35**.

**Scheme 9:**
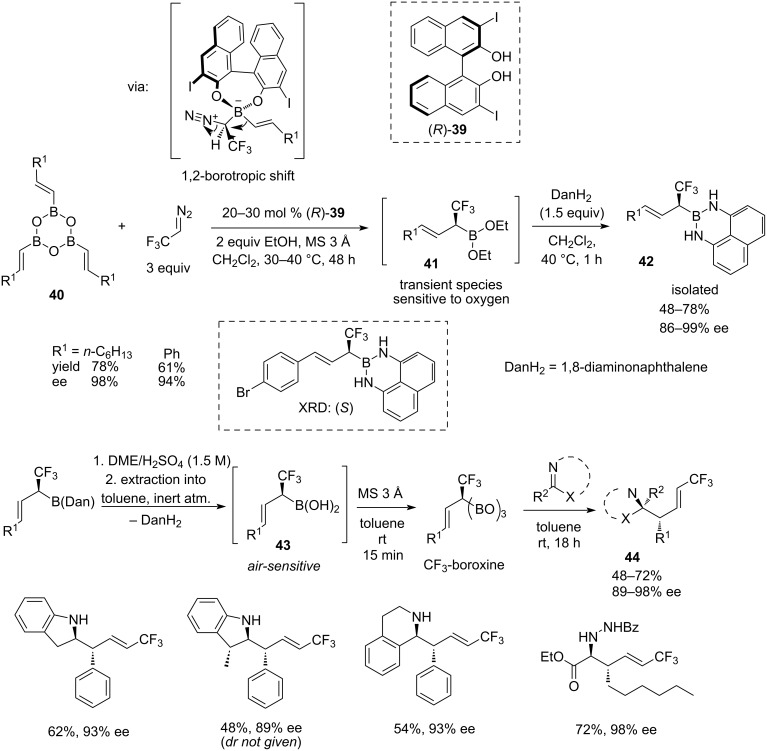
(*E*)-Stereospecific asymmetric α-trifluoromethylallylation of cyclic imines and hydrazones [31].

In late 2020, an important method for synthesising enantiopure terminal (*E*)-trifluoromethyl homoallylic amines **44** was described by Szabó and co-workers (Scheme 9) [31]. This methodology is based on the use of enantiopure α-trifluoromethylallylboronic acids **43**, obtained by a 3,3’-diiodo-BINOL **39**-catalysed asymmetric CH(CF_3_)-homologation of dehydrated vinylboronic acids **40** with trifluoromethyl diazomethane at 40 °C. The resulting enantioenriched transient (α-trifluoromethyl)allylboronic acid diethyl esters **41** were converted into the chromatographically stable 1,8-diaminonaphthalene derivatives **42**, which after hydrolysis and extraction into toluene, were reacted with indole, 3-methylindole, 3,4-dihydroisoquinoline, and benzoyl hydrazone ethyl glyoxylate ester to afford terminal (*E*)-trifluoromethyl homoallylic amines **44** with up to 3 adjacent stereocentres with high to excellent enantioselectivities (89–98% ee) and low to moderate yields (48–72%).

The homologation step proceeds via the stereoretentive 1,2-migration of the vinyl group from the tetracoordinated boron to the highly electrophilic carbon of the diazomethane, concerted with the elimination of the nitrogen molecule. The BINOL catalyst **39** forming arylboronate species, enables the shift to occur asymmetrically. In the second step, the hydrolysis of unreactive diaminonaphthalene derivative **42** gives the poorly reactive and highly air-sensitive trifluoromethylallylboronic acid **43**, but after the dehydration forms boroxine with dramatically increased reactivity towards C=N electrophiles. Because of the small steric size of the (BO_2_) group, the reaction is highly selective towards (*E*)-α-trifluoromethyl homoallylic amine **44**, which otherwise is difficult to obtain in a regiospecific manner. It has to be noted though that manipulations with air-sensitive materials **41** and **43** during the reaction sequence result in reduced total yields of 29–56%. Also, the reaction scope is currently represented by only 4 examples.

### Asymmetric allylation with organosilicon and organotin-based reagents

**Scheme 10:**
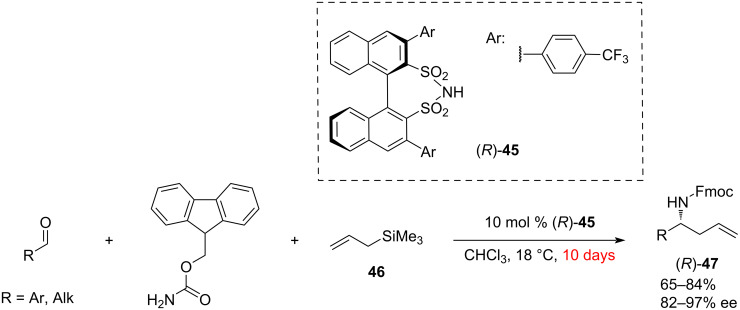
Hosomi–Sakurai-type allylation of in situ-formed *N*-Fmoc aldimines [32].

The first example of a successful organocatalytic enantioselective Hosomi–Sakurai reaction of imines using allyltrimethylsilane was reported by List and co-worker [32]. In this approach, the direct synthesis of Fmoc-protected homoallylic amines **47** was achieved by a three-component coupling of allyltrimethylsilane (**46**) with the in situ-formed *N*-Fmoc-aryl- and -alkylimines, catalysed by a chiral disulfonimide **45** (Scheme 10).

**Figure 1:**
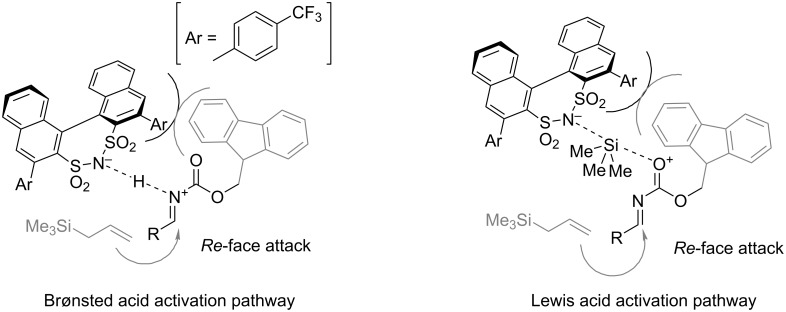
Two different pathways for the Hosomi–Sakurai reaction of allyltrimethylsilane with *N*-Fmoc aldimines.

Since allyltrimethylsilane (**46**) belongs to the type 2 allylation reagents [33], the nucleophilic addition proceeds via an open transition state (Figure 1). Two possible mechanistic pathways were proposed, where the Fmoc-imine is activated either with a Brønsted or Lewis acid. In both cases, the allyl group of the silane reagent **46** attacks from the *Re*-face of the imine, leading to the major (*R*)-enantiomer of **47**. Based on the experimental data obtained with the preformed silylated catalyst and preformed imine, the Lewis acid activation mode appears to be the most plausible.

Interestingly, chiral phosphoric acids failed to catalyse this reaction, but the use of chiral sulphonimide **45** afforded 82–97% ee and 65–84% yield on a set of aromatic and aliphatic aldehydes. The low reactivity of the allyltrimethylsilane (**46**) required an extended reaction time (10 days). The relatively low activity of the sulphonamide catalyst **45** also necessitates higher loadings of 10 mol % (Scheme 10).

**Scheme 11:**
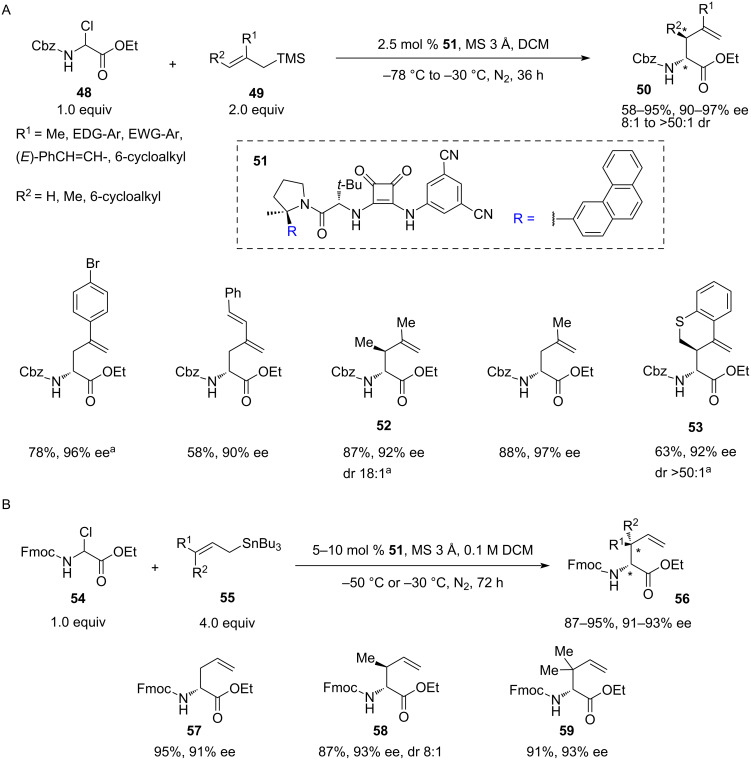
Chiral squaramide-catalysed hydrogen bond-assisted chloride abstraction–allylation of *N*-carbamoyl α-chloroglycinates with trimethylsilane and tri-(*n*-butyl)stannane reagents [34]. ^a^The reaction was conducted at −5 °C.

In 2019, a novel catalytic approach to homoallylic *N*-carbamoylamino esters **50** was described by Jacobsen and co-workers [34]. This allylation method involves squaramide **51**-catalysed chloride abstraction from readily accessible *N*-carbamoyl α-chloroglycinates **48** or **54** (Scheme 11) [35], with a simultaneous nucleophilic attack of allylsilane **49** (Scheme 11A) or allylstannane **55** (Scheme 11B). The reactions are highly sensitive to moisture and oxygen. Therefore, it was conducted under an inert atmosphere in anhydrous dichloromethane with 3 Å molecular sieves.

Benzyl carbamates **48** showed excellent enantioselectivities (90–97%) and moderate to high yields (58–94%) across a wide range of allylation reagents **49** on a submillimolar scale, including 2-methylcrotylsilane (product **52**) and disubstituted cyclic silanes (such as the silane, leading to **53**), locked in *syn*-conformation. For the 2,3-disubstituted allylation products such as **52** and **53**, the diastereomeric ratio spanned across 18:1 to >50:1. Notably, all 2,3-disubstituted silanes required an increased reaction temperature (–5 °C).

A slightly different protocol was elaborated for *N*-Fmoc carbamates **54** using the same catalyst **51**. Instead of allylsilanes **49**, stannanes **55** were employed and the method proved effective in simple allylation, prenylation, and crotylation (**57**–**59**). The yields were also generally higher (87–95%) than with silanes. However, the reaction required an increased loading of stannanes **55** (3.0 or 4.0 equiv) and an increased reaction time (72 vs 36 hours). Also, higher loadings of catalyst **51** (5–10 mol %) were required to achieve the same level of enantioselectivity. It is noteworthy that selected recrystallised products showed 98–99% ee. Importantly, both silane and stannane-based protocols were successfully performed on a 5 mmol scale using substrates **52** and **59**. There was a slight loss in yield and a negligible decrease in stereoselectivity in the case of 2,3-dimethylallylation product **52**.

The limitations in the scope of this methodology include a poor stereoselectivity achieved with 5-membered cyclic 2,3-disubstituted silanes in contrast to the 6-membered analogues (**53**). A lower efficiency was recorded with 2-substituted allylsilanes bearing either a halogen or an alkyl group with terminal halogen or acetate. The 2-ethynylallylsilane decomposed in the reaction conditions, while simple allyltrimethylsilane was unreactive. Among allylstannanes, the (3-phenylallyl)tri-*n*-butylstannane was also unreactive. Interestingly, both tetraallylsilane and tetraallylstannane gave racemic products.

**Figure 2:**
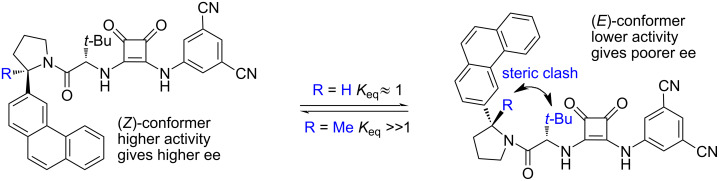
The pyrrolidine unit *gem*-methyl group conformational control in the squaramide-based catalyst [34].

Optimisation of the catalysts structure revealed that the 3-phenanthrylpyrrolidine derivative provided superior yields and selectivities. Notably, the isomeric 9-phenanthrylpyrrolidines remained selective but gave noticeably lower yields. The methyl group, geminal to the phenanthryl, plays an important part in securing the enantioselectivity of the reaction by locking the catalyst in the more active and enantioselective *Z*-configuration (Figure 2).

Further, the authors found that H-bond donors featuring urea, thiourea, and guanidine motifs were either inactive or provided racemic mixtures. Among 3,5-dichloro-, 3,5-di(trifluormethyl)- and 3,5-dicyanophenylsquaramides, the latter proved to be the most active but all three provided a similar enantioselectivity.

**Figure 3:**
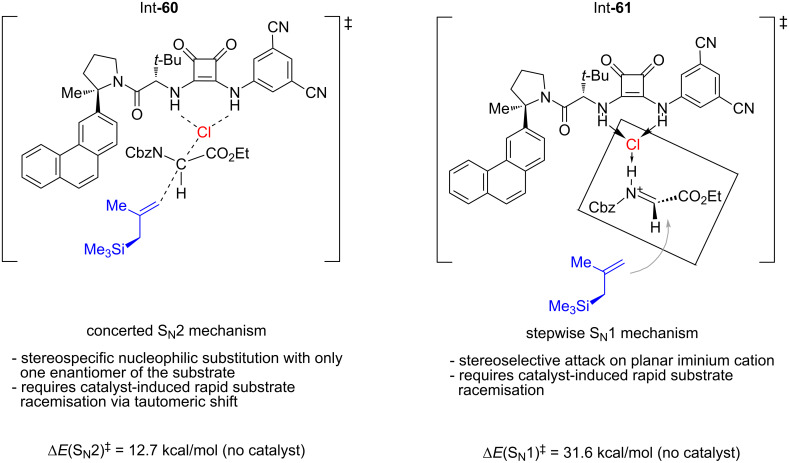
The energetic difference between the transition states of the two proposed modes of the reaction (S_N_1 and S_N_2 mechanisms). The concerted S_N_2 mechanism is energetically more favoured [34].

Kinetic studies revealed that the reaction displayed the 1st order in both the allyl reagent and the glycinate substrate, which may indicate either a concerted S_N_2 mechanism or S_N_1 mechanism with the allyl nucleophile addition step as rate-determining. Interestingly, the authors observed a 0.55 order in the catalyst, which indicates that a substantial part of the catalyst forms a hydrogen-bonded dimer with *K*_diss_ ≈ 1000 M^−1^ when not in the catalytic cycle, an observation that is in good agreement with previously obtained data on bisurea catalysts [36]. To further distinguish between the S_N_1 and S_N_2 mechanisms, the authors performed an in silico simulation of the non-catalysed reaction to determine the possible potential energy surface and found that concerted S_N_2-key intermediate **60** must be at least 18.9 kcal/mol more favoured than a separated imine–chloride ion pair **61** attacked by the free allylsilane (Figure 3).

Altogether, the developed methodology can be formally viewed as a useful tool for the enantioselective synthesis of chiral α-carboxyl-2,3-disubstituted homoallylic amines **50** and **56** (Scheme 11). However, the high sensitivity of silanes **49** to air and moisture along with an increased toxicity of organotin compounds **55** may be considered challenging factors in its application on a larger scale.

**Scheme 12:**
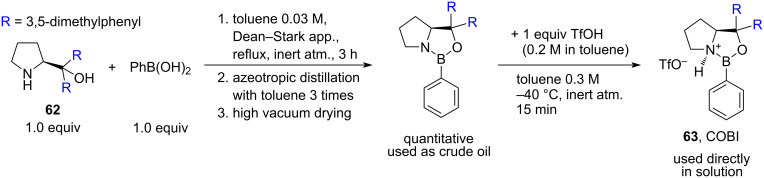
One-pot preparation procedure for oxazaborolidinium ion (COBI) **63** [37].

**Scheme 13:**
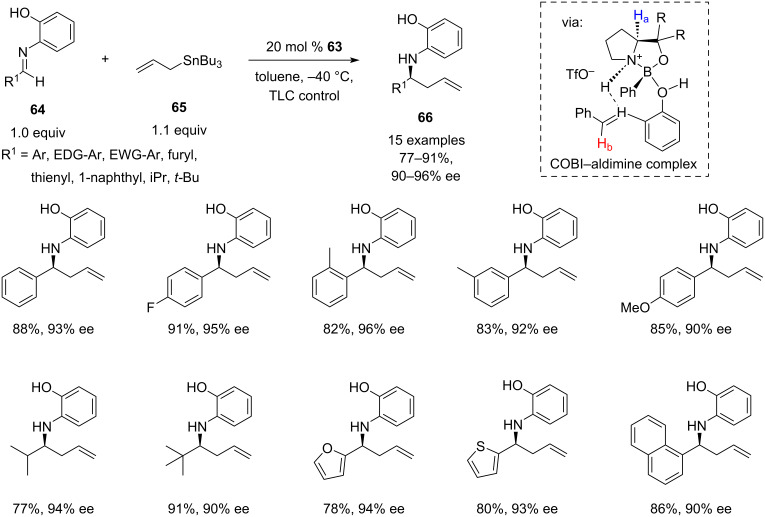
Chiral oxazaborolidinium ion (COBI)-catalysed allylation of *N*-(2-hydroxy)phenylimines with allyltributyltin. Scope of the reaction [37].

In 2019, an interesting approach to the organocatalytic enantioselective allylation of imines was described by Ryu’s group as a part of a wider asymmetric nucleophilic addition methodology [37]. The method is based on the use of 20 mol % of sterically hindered chiral oxazaborolidinium ion (COBI) **63**, that can be readily prepared from a relatively inexpensive commercially available derivative of ʟ*-*proline, (*S*)-(−)-α,α-bis(3,5-dimethylphenyl)-2-pyrrolidinemethanol (**62**) (Scheme 12). This catalyst is capable of asymmetric activation of *N*-(2-hydroxyphenyl)imines through the reversible chelation to the *N*-(2-hydroxyphenyl) group, forming a rigid intermediate, while the aryl group of the oxazaborolidinium ion is restricting the allyltributylstannane attack to only one enantioface of the imine C=N bond (Scheme 13, COBI–aldimine complex). The catalyst was used in the allylation of a wide range of aliphatic and aromatic *N*-(2-hydroxyphenyl)imines **64**, providing good to excellent yields (77–91%) and excellent enantioselectivities (90–96%) on a millimolar scale (Scheme 13).

**Scheme 14:**
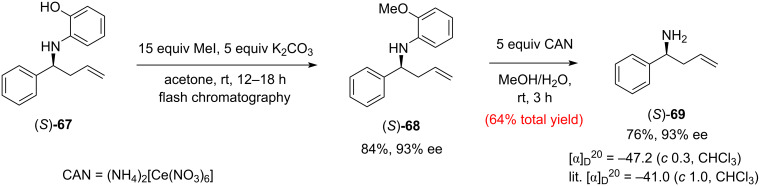
The two-step *N*-(2-hydroxy)phenyl group deprotection procedure [37].

It is worth noting the use of optimised minimal excess of allyltributyltin (**65**), which to some extent addresses the higher toxicity of trialkylstannanes compared to their silicon counterparts. The absolute configurations of homoallylamines **66** were assigned by analogy to **67**, the configuration of which was determined after removal of the *N*-aryl group and comparing the optical rotation with the literature values for the known enantiomer (*S*)-**69** (Scheme 14).

The proposed stereochemical model suggests, that the 2-aminophenol functionality in the substrates is a prerequisite for attaining high enantioselectivity in the allylation. Interestingly, the aldimines synthesised from 2-hydroxy-4-methylaniline produced inferior yields. While the *N*-aryl homoallylic amines **66** can be useful intermediates in total synthesis, the deprotected derivatives such as **69** are desired for the synthesis of natural products. Therefore, an easy-to-perform two-step deprotection procedure was developed that is based on methylation of the phenolic hydroxy group with an excess of MeI in acetone at rt (**68**), followed by oxidative cleavage of the methoxyphenyl group in aqueous methanol using excess of ceric ammonium nitrate (CAN) as a soft oxidant (Scheme 14). The sequence afforded the target (*S*)-homoallylic amine **69** in 64% overall yield with a complete retention of chirality.

**Scheme 15:**
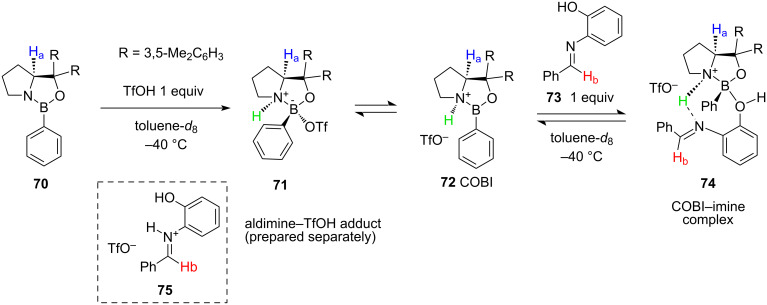
Low-temperature (−40 °C) NMR experiments evidencing the reversible formation of the active COBI–imine chiral complex [37].

To gain a mechanistic insight into the formation of the active catalytic species in COBI-catalysed allylations, chemical equilibria in a solution containing catalyst **72**, triflic acid, and aldimine **73** were investigated by low-temperature NMR spectroscopy (Scheme 15).

Protonation of oxazaborolidine **70** with triflic acid resulted in an 8.3:1 mixture of **71** and **72**, the latter acting as a catalytically active species. After the addition of 1 equiv of imine **73** at −40 °C, proton H_a_ of **72** shifted upfield which was close to what was observed in the TfOH adduct **71**, and which supported the formation of intermediate **74**. The authors estimated the equilibrated ratio between COBI **72** and coordinated COBI–imine complex **74** as 1 to 4.6. Despite the low accuracy of this estimate, it is clear that both species **72** and **74** are present, and that coordination is reversible under the reaction conditions. The formation of the COBI–imine complex was further evidenced by comparing chemical shifts of the H_b_ proton in **74** with the same signals in the free substrate **73** and protonated **75** at −40 °C. It was revealed that the aldimine proton H_b_ in the free imine appears as a singlet at 7.95 ppm, while in both **74** and **75** it becomes a doublet with a 15 Hz *trans J*_H–H_ coupling constant. However, in the COBI–imine complex **74** it appears at 8.41 ppm, while in the imine TfOH salt **75** it shows at 9.24 ppm.

**Figure 4:**
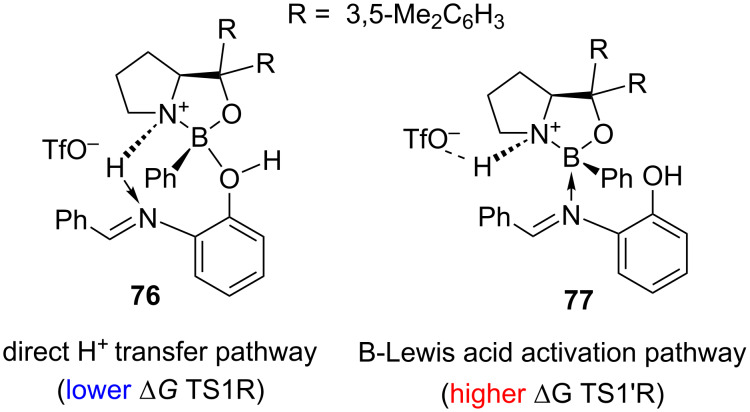
Two computed reaction pathways for the COBI-catalysed Strecker reaction (TS1 identical to allylation – COBI–imine complex) reported by Chen and Qiao in 2023 [38].

This detailed NMR investigation sparked further interest in the structure of the transition state of this reaction. An analysis of the potential energy profiles of the two hypothetical pathways of the analogous nucleophilic addition of cyanide was reported by Chen and Qiao [38] in 2023 (Figure 4).

The calculations revealed that Δ*G* profile for the reaction pathway via **76**, in which the imine molecule is activated by the direct chiral proton transfer from the COBI ion and then later attacked by a nucleophile, is energetically favoured over pathway via **77**, in which the imine is activated directly by coordination to the Lewis acidic boron atom of the free imine complex. This supports the mechanism proposed in the original study based on the NMR experiments.

**Scheme 16:**
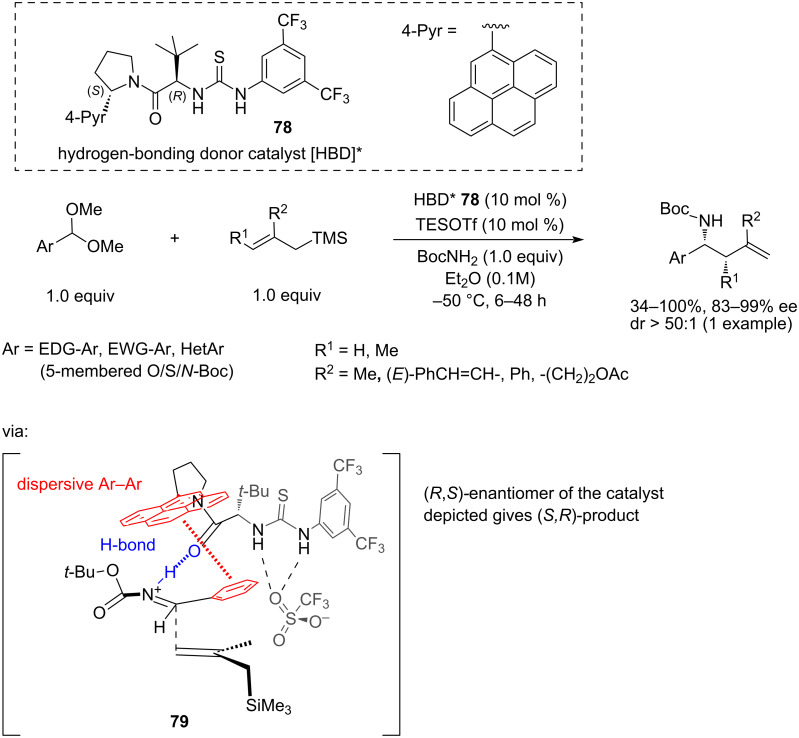
Highly chemoselective and stereospecific synthesis of γ- and γ,δ-substituted homoallylic amines by a three-component reaction between an aryl acetal, BocNH_2_ carbamate and a 2,3-disubstituted allylsilane, in presence of the triflate-assisted bifunctional HBD/π_Ar-Ar_ catalyst [39].

In 2021, Jacobsen and co-workers [39] developed a direct, highly enantioselective three-component reaction between aromatic acetals, BocNH_2_, and various 2-monosubstituted or 2,3-disubstituted allyltrimethylsilanes in the presence of 10 mol % of triethylsilyl triflate (TESOTf) and 10 mol % of the chiral bifunctional thiourea catalyst **78** with a pending 4-pyrenyl group. The catalyst acts as H-bond donor but additionally it controls the chiral environment through π-stacking interaction with the aryl group of the substrate, blocking one of the enantiofaces of the iminium intermediate **79**. The reaction proceeds in Et_2_O at −50 °C and requires 18 hours (Scheme 16).

**Scheme 17:**
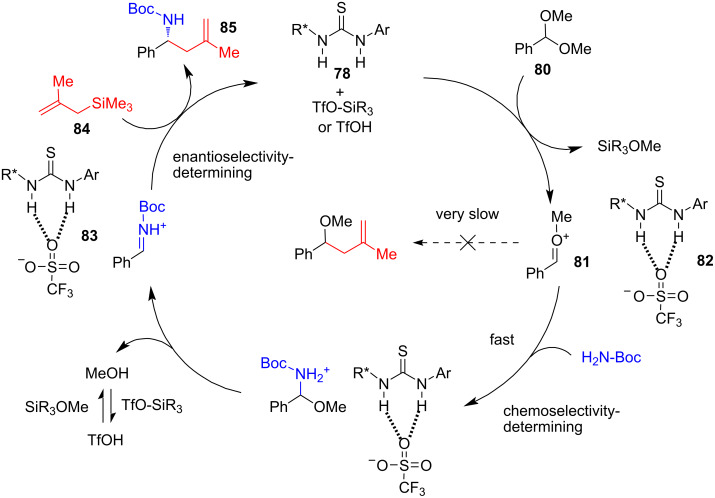
Catalytic cycle for the three-component allylation with HBD/π_Ar–Ar_ catalyst [39].

A proposed catalytic cycle is shown in Scheme 17. In this reaction, the aromatic acetal **80** gives rise to a hydrogen-bonded triflate **82** and oxocarbenium ion **81**. The latter quickly reacts with BocNH_2_ forming the ArCH=HN^+^-Boc iminium–triflate ion pair **83**. Notably, the attack of the C-nucleophile on **81** is very slow, which determines the chemoselectivity of the reaction. Complex **83** of the iminium ion and the catalyst then reacts with allylsilane **84** through an open, type 2 [33] transition state from the exposed enantiotopic face, as in int-**79**, to afford product **85** and to release catalyst **78** completing the catalytic cycle (Scheme 17).

**Scheme 18:**
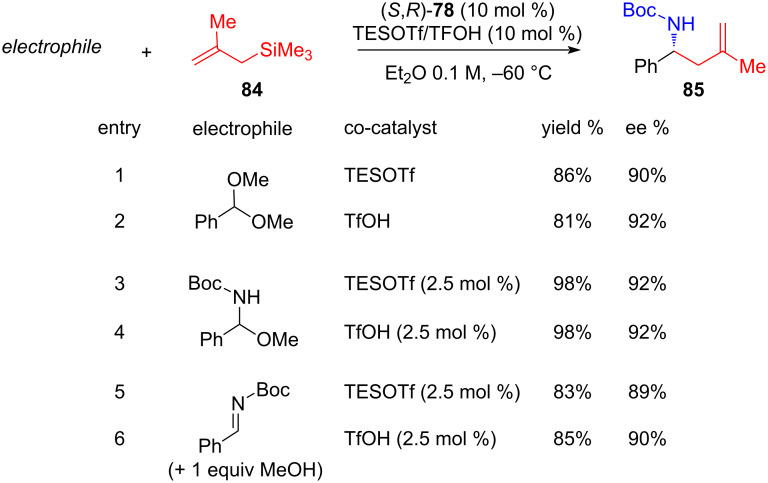
Reactivity of model electrophiles [39].

In a mechanistic probe, the proposed reaction intermediates were used as starting electrophiles under the standard reaction conditions (Scheme 18). In all three cases, identical outcomes were achieved, supporting the proposed catalytic cycle.

**Scheme 19:**
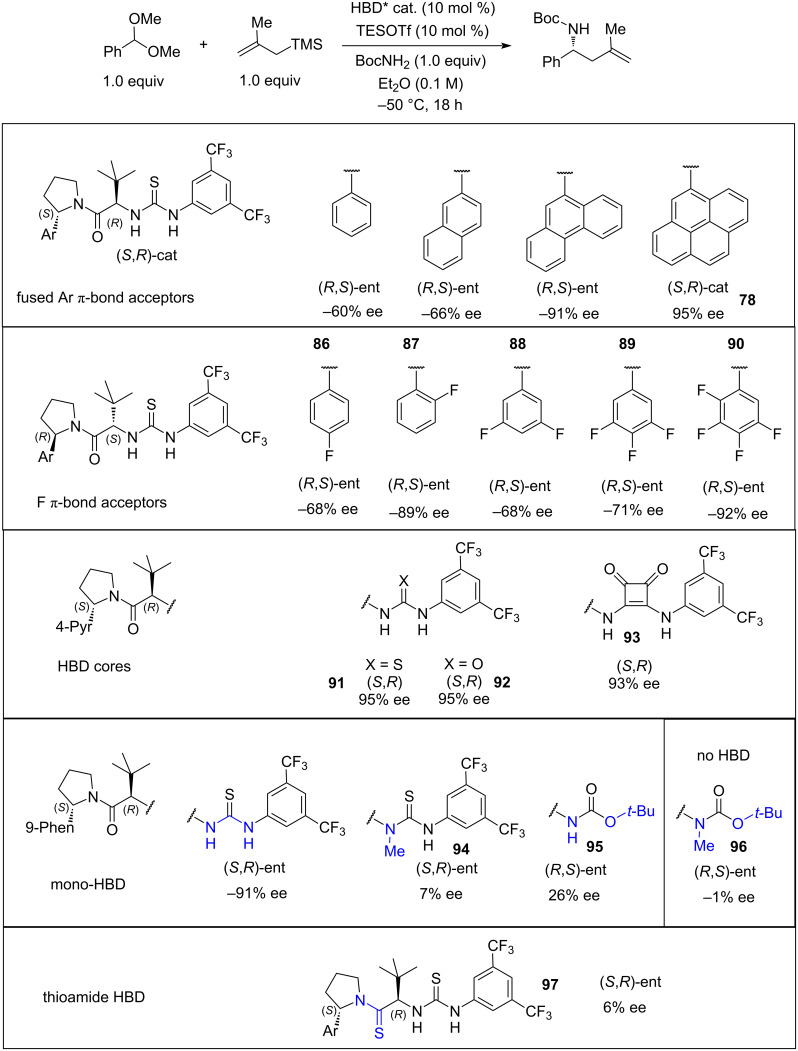
HBD/π_Ar–Ar_ catalyst rational design and optimisation [39].

In the optimisation of the catalyst **78** structure, several important observations were made (Scheme 19). Replacing thiourea (**91**) as the H-bond donor unit with squaramide (**93**) or urea (**92**) did not affect the enantioselectivity of the allylation of the benzaldehyde-derived acetal. On the other hand, blocking (**94**) or removing (**95**) one of the N–H groups responsible for H-bonding resulted in a dramatic loss of enantioselectivity (7% ee in **94** and 26% ee in **95**), while removing both of the N–H groups gave the racemate (1% ee for **96**). Interestingly, thioamide **97** also showed a low ee. Replacing the 3,5-bis(trifluoromethylphenyl) group with other fluorinated aromatics (**86**–**90)** showed a gradual decrease in ee (92% ee in **90** to 68% ee in **86** and **88**) depending on the degree of fluorination. Computational modelling for the 3,5-bis(trifluoromethylphenyl) catalyst **78** revealed that π-stacking interactions of the 4-pyrene group with the aromatic group of the substrate had a significant contribution to the stereoselectivity of the reaction.

**Scheme 20:**
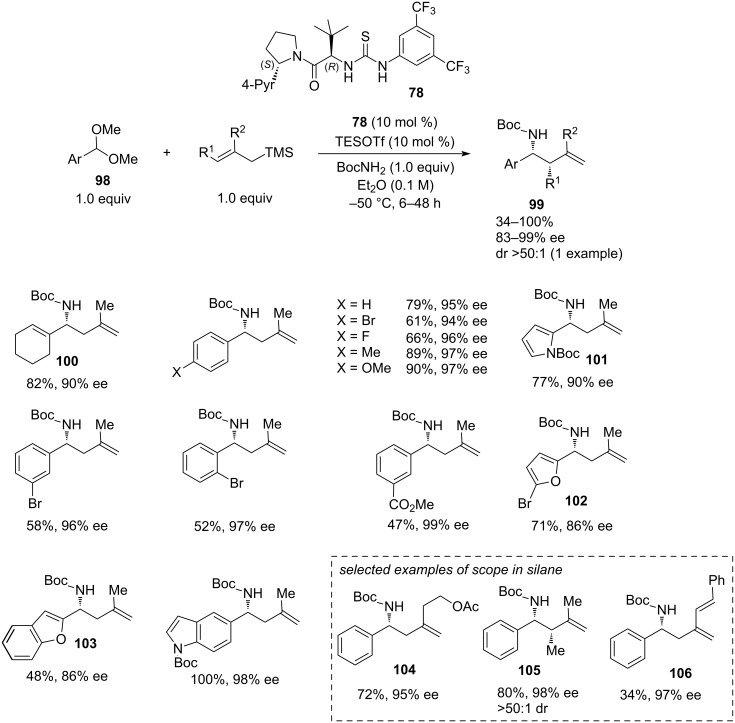
Scope of the three-component HBD/π_Ar–Ar_-catalysed reaction [39].

The reaction demonstrated a broad scope both in the acetal **98** and in the allylsilane (Scheme 20). A high enantioselectivity was achieved even with the non-aromatic 1-cyclohexene-1-carboxaldehyde-derived acetal **100** (82% yield, 90% ee). 2-Methallylsilane showed universally high enantioselectivity across a range of acetal components (95–99% ee), except for a slight drop in the case of 5-bromo-2-furan (**102**, 86% ee) and 2-benzofuran-substituted (**103**, 86% ee) substrates. Regarding the silane scope, good results were achieved with (*E*)-trimethyl(2-methylbut-2-en-1-yl)silane to give homoallylic amine **105** with 80% yield, 98% ee, and >50:1 dr. The reactions of benzaldehyde dimethyl acetal with (2-[2-(acetoxy)ethyl]allyl)trimethylsilane (producing **104**) and with 2-(cinnamyl)allyltrimethylsilane (producing **106**) also showed high enantioselectivity, although the yields varied from modest to good (Scheme 20).

**Scheme 21:**
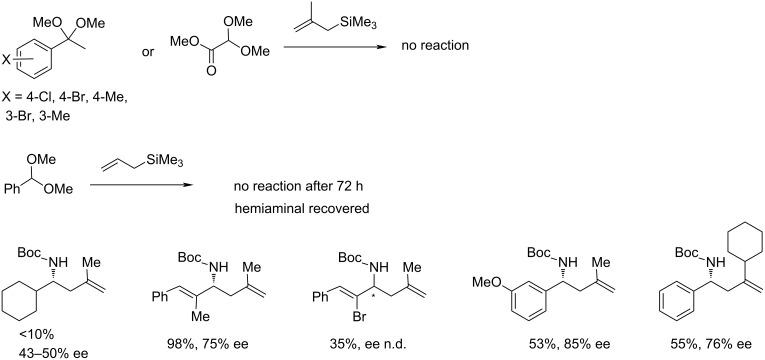
Limitations of the HBD/π_Ar–Ar_-catalysed reaction [39].

Limitations of the scope include the inertness of ketals and methyl dimethoxy acetal (glyoxalic acid derivative) in this reaction. Also, allylation with a simple allyltrimethylsilane failed to give the desired product **99** after 72 h returning hemiaminal (Scheme 21). Alkyl acetals, such as cyclohexyl carboxaldehyde acetal reacted sluggishly with low yield and selectivity (<10% yield, 43–50% ee), possibly due to their inability to participate in π–π interactions. In general, the π-stacking interaction between the catalyst and the aryl group of the substrate in the enantio-determining step appears to affect the scope. Other problematic substrates included (*Z*)-α-methylcinnamyl and (*Z*)-α-bromocinnamyl acetals giving the respective products with lower ee. Despite some phenyl substrates with 3-substitution (CO_2_Me, OCH_2_, Br) showing excellent enantioselectivity, the 3-methoxyphenyl-substituted substrate afforded the homoallylic amine in only 85% ee. The bulky 2-cyclohexylallyltrimethylsilane showed only moderate yield and enantioselectivity (55%, 76% ee).

**Scheme 22:**
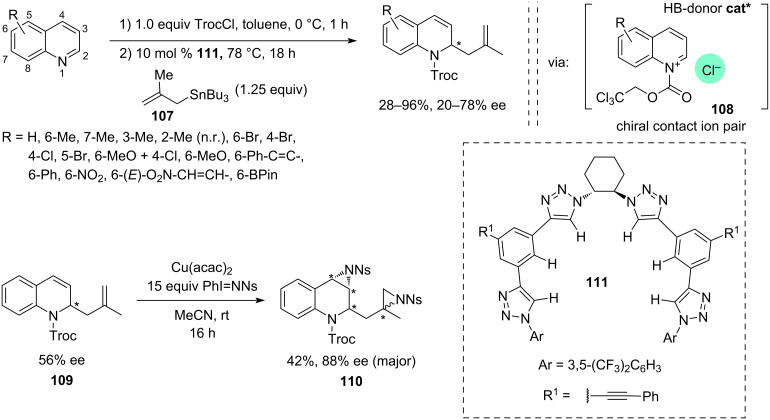
Asymmetric chloride-directed dearomative allylation of in situ*-*generated *N*-acylquinolinium ions, catalysed by a tetrakis-triazole HBD catalyst [40].

In 2022, Mancheño and co-workers [40] exploited the application of hydrogen-bond donor catalysis in asymmetric dearomative α-allylations of in situ-generated *Ν*-acylquinolinium salts **108** generated with 2,2,2-trichloroethyl chloroformate (TrocCl) (Scheme 22). Screening through a range of catalysts including the known squaramide and thiourea analogues, a novel tetrakis-triazole-based H-bond donor catalyst **111** was identified as most promising. Among different nucleophilic allylating reagents, 2-methallyltributyltin (**107**) emerged as optimal in terms of reactivity and enantioselectivity. It was speculated that the enantioinduction is realised via the H-bond-donor association of catalysts **111** to the Cl^−^ ion of the *N-*acylquinolinium intermediate **108**, though a detailed investigation into the mechanism of the enantiodifferentiation has not been carried out (Scheme 22).

In total 15 different quinolines were tested. The yields varied in the range of 28–96%, whereas enantioselectivities remained modest (20–78% ee). To illustrate the synthetic utility of the products, a double Cu(acac)_2_-catalysed aziridination of the model substrate **109** was carried out using a large excess of PhI=NNs reagent (15 equiv, Ns = nosyl) to afford diastereoselectively diaziridine **110** with 88% ee albeit with a modest 42% yield. Overall, this is an interesting example of dearomative allylation of *Ν*-acylquinolinium salts, though enantioselectivity currently is too low to ensure wider practical application of the method.

### Enantioselective 2-aza-Cope rearrangement

**Scheme 23:**
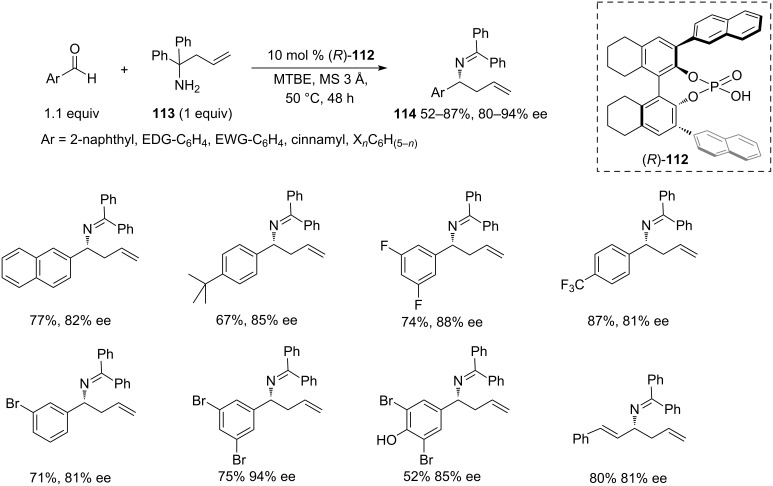
Chiral phosphoric acid-catalysed aza-Cope rearrangement of in situ-formed *N*-α,α’-diphenyl-(α’’-allyl)methyliminium cations reported by Rueping et al. 2008 [41].

In 2008, a conceptually different methodology was reported by Rueping and co-workers [41] that was based on the aza-Cope rearrangement of in situ-formed *N*-α,α’-diphenyl-(α’’-allyl)methyliminium cations catalysed by the BINOL-derived chiral phosphoric acid **112** (Scheme 23). The amine **113** acting as the allyl donor source was obtained by the addition of allylmagnesium bromide to benzophenone imine on a 3 gram scale in 80% yield. The rearrangement products **114** were readily converted to free homoallylic amines employing hydroxylamine in THF.

The reaction showed moderate to high yields on various aromatic aldehydes, being slightly more efficient with electron-poor substrates. On the other hand, high enantioselectivity was maintained over the aldehyde scope regardless of steric size and position of the substituents.

**Scheme 24:**
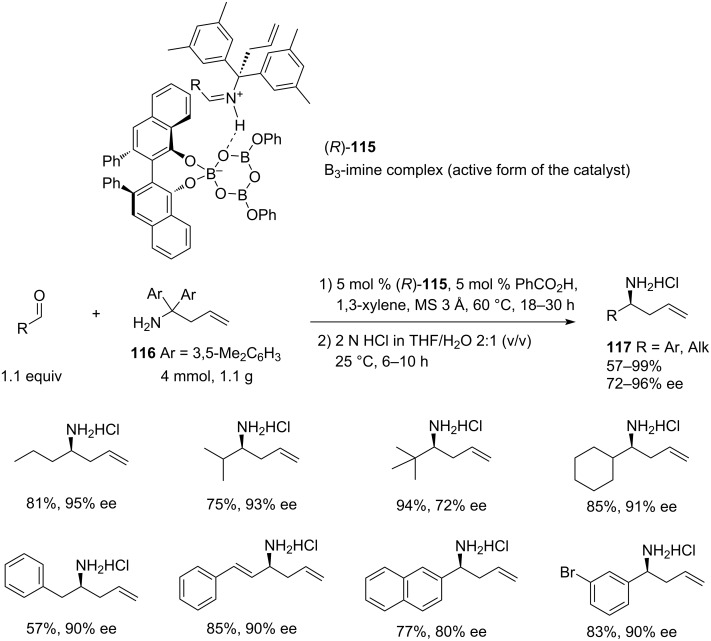
Tandem (*R*)-VANOL-triborate-catalysed asymmetric aza-Cope rearrangement of in situ-formed aldimines using allyl transfer reagent **116** [42].

A few years later, Wulff and co-workers [42] further elaborated the aza-Cope rearrangement methodology by employing a bulkier allyl-transfer reagent **116**. They used a novel pre-formed chiral triborate catalyst **115** in tandem with a non-chiral Brønsted acid and expanded the scope to aliphatic homoallylic amines (Scheme 24). The new method established a scalable, chromatography-free purification protocol for the synthesis of homoallylic amines **117** and showed increased yields and enantioselectivities for aromatic aldehydes. In addition, a simple, ambient-temperature hydrolysis procedure was reported that yielded the unprotected analytically pure homoallylic amines in high yields without the need for chromatographic purification. The bulky aryl groups in **116** were synthesized in a one-pot procedure, starting from 3,5-dimethylbenzonitrile, by the addition of 3,5-dimethylphenylmagnesium bromide and allylmagnesium chloride (24% combined yield on a 1 g scale). The authors also optimised the protocol for a large-scale synthesis of the allyl donor reagent (up to 25 g). Importantly, benzophenone was easily recovered by recrystallisation after the workup (no chromatography, 80% yield) and then converted back into the allyl donor **116** (86% yield), thus boosting the overall atom-economy of the process. Also, the recovery of the (*R*)-VANOL ligand **115** was attained by column chromatography in 92%.

**Scheme 25:**
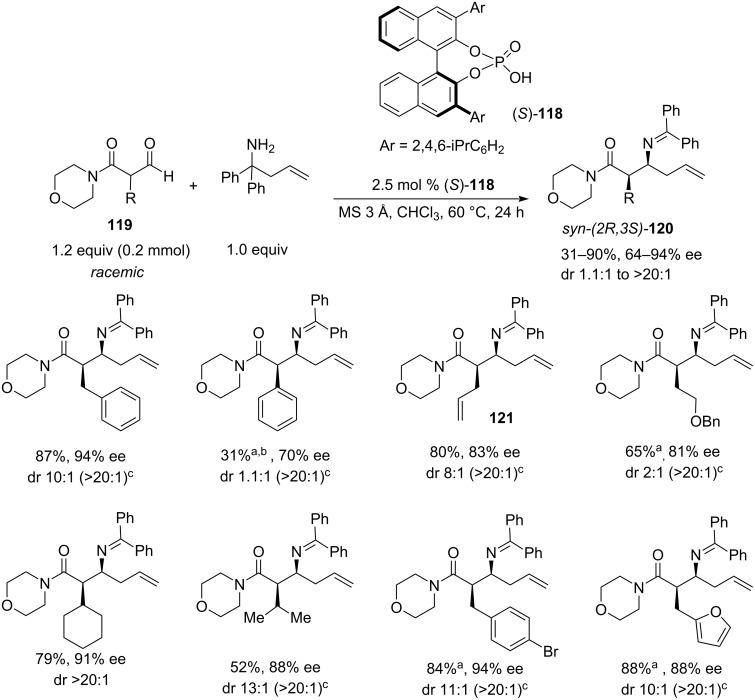
(*S*)-TRIP-catalysed enantioconvergent aza-Cope rearrangement of β-formyl amides, substrate scope [43]. ^a^The yield was measured after hydrolysis of the benzophenone imine. ^b^The reaction required 300 W microwave irradiation at 130 °C for 6 hours. ^c^Isolated dr.

In 2015, further development of the 2-aza-Cope rearrangement strategy was reported by Johnson [43]. This work expanded the scope of the reaction to β-formyl amides **119** under the conditions of dynamic kinetic resolution (DKR) by employing chiral Brønsted acid catalyst (*S*)-TRIP (**118**) (Scheme 25). In this approach, the racemic β-formyl amide forms the iminium intermediate that undergoes fast equilibration via the enamine tautomer to form preferentially one enantiomer which then undergoes the acid-catalysed aza-Cope rearrangement, creating the desired *syn*-(2*R*,3*S*)-homoallylic amine **120** in high diastereo- and enantioselectivity.

**Scheme 26:**
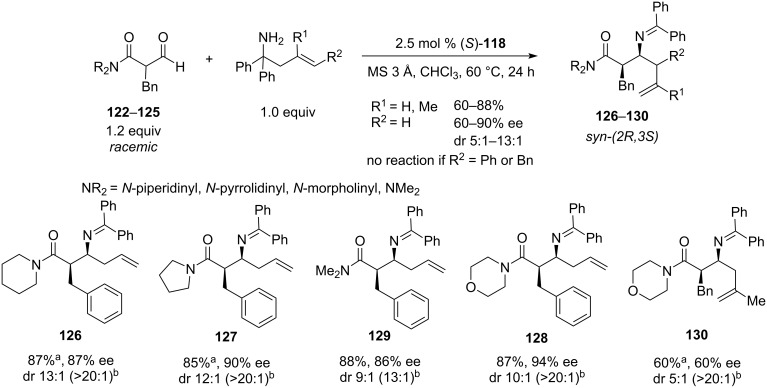
(*S*)-TRIP-catalysed enantioconvergent aza-Cope rearrangement of β-formyl amides **16**–**19**, amide and allyl group scope [43]. ^a^The yield was measured after the benzophenone imine hydrolysis. ^b^The isolated dr.

The reaction was tested with various aryl, benzyl, allyl, and alkyl-functionalised β-formyl *N-*morpholinyl amides **119**; the amide function was also varied. Yields were scattered across the 31–90% range, with the diastereoselectivities varying from 1.1:1 to >20:1 (Scheme 25). Interestingly, almost all isolated products, except for the amine resulting from methyl β-formyl *N-*morpholinyl amide (**119**, R = Me), showed dr > 20:1. Enantioselectivity followed the trend and varied between 64–94%, the lowest being obtained for the methyl-substituted substrate **119** and the highest for bulky benzyl substrates. Several reactions showed 94% ee which appears to be an upper limit for this particular (*S*)-TRIP-catalysed reaction. Screening the amide group revealed, that *N*-morpholinyl (**122**), *N*-piperidinyl (**123**), *N*-pyrrolidinyl (**124**), and *N,N*-dimethyl (**125**) amides produced the corresponding homoallylic amines **126**–**130** with dr ≥ 9:1. In the case of R = Bn, all these amides showed yields greater 85% and ees above 86% for the unsubstituted allyl group transfer (**126**–**129**). The reaction showed low tolerance to changes in the allyl fragment: with a methallyl derivative **130**, the stereoselectivity dropped to a modest level (dr 5:1, ee 60%, yield 60%), whereas higher allyl homologues such as phenyl and benzyl proved to be inactive (Scheme 26).

**Scheme 27:**
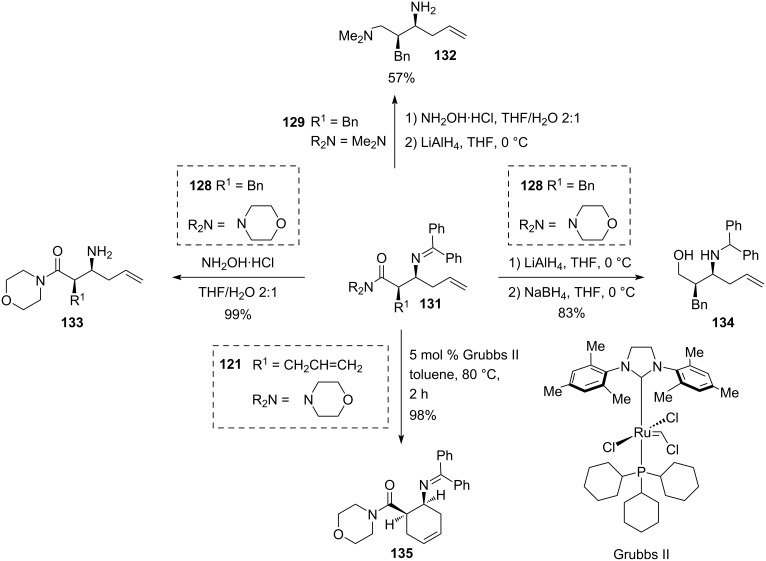
Synthetic applications of homoallylic *N*-benzophenone imine products **131** [43].

The synthetic utility of the resulting homoallylic *N*-benzophenone imines **131** was illustrated on a laboratory scale (Scheme 27). Thus, the hydrolysis of the N=CPh_2_ group in the *N*-morpholinyl product **129** with hydroxylamine hydrochloride afforded the deprotected homoallylic amine **133** in a 99% yield without need for chromatographical purification. In a separate experiment, the sequential reduction of the *N*-morpholinyl amido and benzophenone imino groups with LiAlH_4_ and NaBH_4_ afforded the corresponding *syn*-2-benzyl-1,3-amino alcohol **134** in 83% yield. In the case of the dimethylamide derivative, deprotection of benzophenone imine **128** with NH_2_OH·HCl followed by the LiAlH_4_ reduction gave rise to chiral *syn*-2-benzyl-1,3-diamine **132** in 57% yield. The ring-closing metathesis of **121** (R^1^ = allyl, from Scheme 25) provided pure *N*-cyclohexenyl imine **135** in 98% yield.

### Asymmetric alkylation of imine-carbanion

**Scheme 28:**
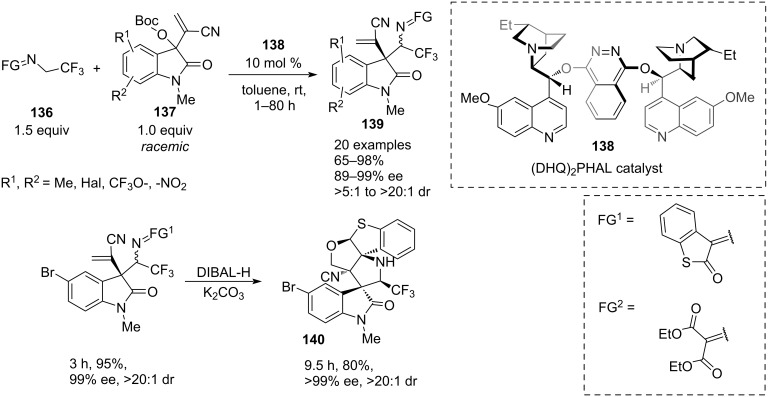
Chiral organocatalysed addition of 2,2,2-trifluoroethyl ketimines to isatin-derived Morita–Baylis–Hilman carbonates [44].

In 2023, a team of Huang and Yan [44] presented a novel approach towards synthetically important homoallylic α-trifluoromethylamine derivatives of high molecular complexity (Scheme 28). The process involves a highly enantioselective reaction of the isatin-derived Morita–Baylis–Hillman carbonate **137** with a novel α-CF_3_-substituted imine **136**, derived from inexpensive benzothiophene-2,3-dione. A *C*_2_-symmetrical cinchona-derived (DHQ)_2_PHAL catalyst **138** has been identified as the most efficient that afforded high yield and enantioselectivity with a 10 mol % loading. The diastereoselectivity was 20:1 or better in most cases and was found to be independent of the reaction conditions.

The optimised protocol involved stirring 1 equiv of isatin with a 50% excess of the α-CF_3_-substituted imine in toluene at room temperature in the presence of 10 mol % of organocatalyst **138** for times between 1 and 80 h. The reaction exhibited a broad scope in the isatin carbonate derivatives with high to excellent enantioselectivities (89–99%), good to excellent yields (65–98%), and consistently high diasterioselectivities (>20:1). For selected examples, the reaction was successfully performed on a 1 gram scale. Apart from benzothiophene-2-one imine, the reaction also worked well with diethyl oxomalonate-derived trifluoromethylimine.

A practical potential of the methodology was illustrated by constructing the medicinal chemistry-relevant highly fused spiro compound **140** featuring 5 stereocentres (3 quaternary) in 99% ee and 76% overall yield from achiral **136** and racemic **137** in just 2 steps. Selected substrates were converted to the corresponding free amines by hydrolysis with aqueous HCl. The reaction proceeded in good yields and with complete retention of stereointegrity.

**Scheme 29:**
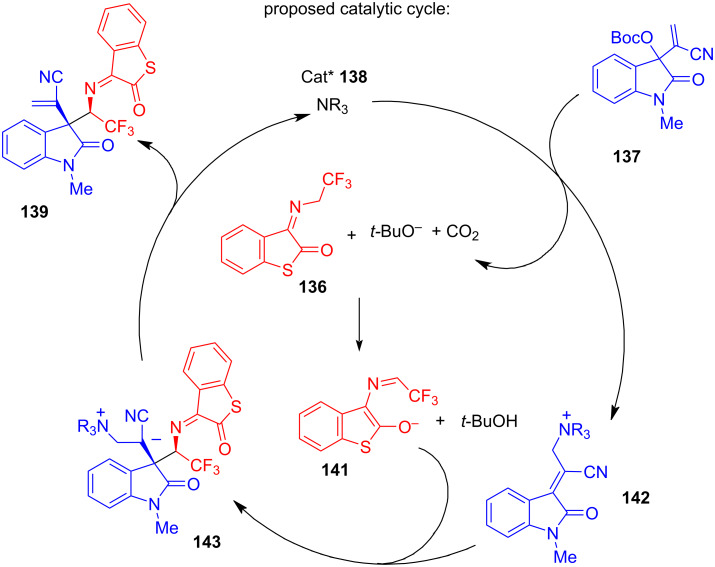
Chiral chinchona-derived amine-catalysed reaction between isatin-based Morita–Baylis–Hilman carbonate and benzothiophene-2-one α-trifluoromethylimine [44].

In a proposed mechanism, isatin carbonate **137** reacts with a quinuclidine unit of the catalyst **138** by an S_N_2’ attack to form cationic intermediate **142**. The *t*-BuO^−^ anion formed in this process abstracts the γ-CH proton of the CF_3_-imine (**136** → **141**), facilitating its nucleophilic attack on the isatin cation (**141** + **142** → **143**), followed by elimination of the catalyst **138**, which completes the cycle liberating **139** (Scheme 29).

Overall, this methodology represents a convenient tool for the rapid construction of medicinal chemistry-relevant heterocyclic homoallylic amines in an enantioselective manner without involving nucleophilic allylation. The highly functionalised products offer the possibility of further derivatisation.

### Other catalytic approaches

**Scheme 30:**
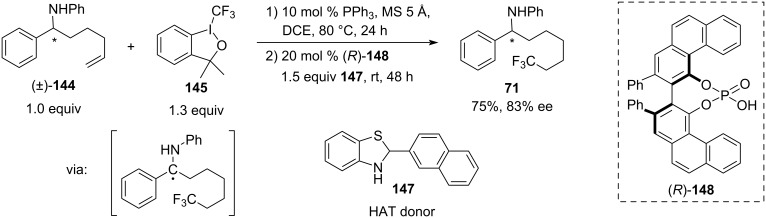
(*R*)-VAPOL-catalysed hydrogen atom transfer deracemisation [45].

In 2017, the deracemisation of an unsaturated amine **144** was reported by Li Dang and Xin-Yuan Liu (Scheme 30) [45]. They used CF_3_-radical-induced remote CH-activation, combined with Brønsted acid-catalysed chiral hydrogen atom transfer (HAT).

In this reaction, triphenylphosphine first mediated the addition of the CF_3_-radical generated from Togni’s reagent (**145**) to a double bond of the δ-alkenylamine, followed by intramolecular hydrogen atom transfer and a single-electron oxidation of the intermediate alkyl radical to form an imine that is then reduced by hydrogen donor **147** catalysed by CPA (*R*)-VAPOL (**148**). The trifluoromethylated amine **146** was obtained in 75% yield and 83% ee. The scope of this reaction is yet to be explored.

**Scheme 31:**
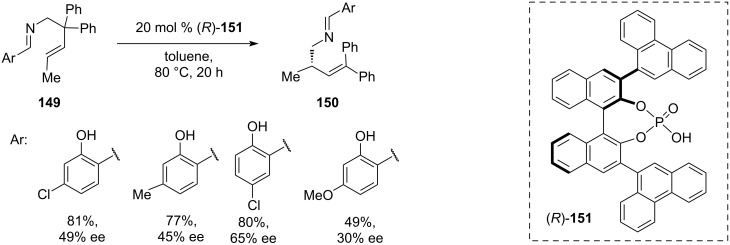
Chiral PA-catalysed [1,3]-rearrangement of ene-aldimines [46].

In 2022, Momiyama and co-workers presented a novel synthetic approach towards enantioenriched homoallylic amines **150** that bear a chiral centre on the β-carbon, based on an asymmetric [1,3]-rearrangement of ene-aldimines **149**, catalysed by the BINOL-derived chiral phosphoric acid (CPA) (*R*)-**151** (Scheme 31) [46]. DFT computational analysis suggested that the reaction proceeds via a complex cascade that involves the fragmentation of ene-aldimine **149** to form an imine methylene cation, which in turn catalyses the methylene group transfer, resulting in azonia-[3,3]-sigmatropic rearrangement, followed by regeneration of methylene imine by an elimination step [47]. The reaction proceeded in toluene at 80 °C with 20 mol % of the CPA catalyst **151** over 20 hours resulting in low to moderate enantioselectivities (30–65% ee) and low to moderate yields (49–81%). However, despite the modest enantioselectivity, the proposed approach presents an appealing strategy towards chiral homoallylic amine scaffolds **150** that are difficult to achieve using other methods.

## Conclusion

Summarising all the analysed approaches, 4 distinct methodological classes can be outlined: (i) Open TS-type [33] Si/Sn reagent-based asymmetric allylations; (ii) closed TS-type [33] allylation of imines with boron-based reagents; (iii) 2-aza-Cope rearrangement methodologies that use achiral *gem*-diaryl homoallylic amines as allyl group transfer reagents; (iv) direct metal-free imine carbanion addition to electrophilic alkene.

Class (i) underwent an evolution from catalysis by covalent interaction to chiral hydrogen-bonded catalysis, which allowed the expansion of the allyl component scope from simple allyl to substituted allyl groups, along with a higher tolerance to imine components. However, one of the unsolved challenges within this class remains the asymmetric allylation of unprotected aldimines. In addition, the use of secondary chiral allylsilanes to access (*Ε*)- or (*Ζ*)-homoallylic amines remains unexplored.

Class (ii) was enriched significantly over the last 2 decades and transitioned from the use of unstable unsubstituted non-cyclic dialkylallylboronates and air-sensitive allylboronic acids in combination with chiral BINOL catalysts to the bench-stable purified linear and branched crotylboronates, their activated boroxine forms, and catalysis by more active chiral phosphoric acids. The catalytic asymmetric allylation of unprotected aldimines remains a challenge. In addition, while the enantioselective and regiospecific methodology towards chiral (*E*)-CF_3_-homoallylic amines has been established, the regiospecific enantioselective approach towards the formation of chiral (*Z*)-homoallylic amines remains unexplored.

Class (iii) attracted the greatest attention in the previous decade mainly due to the absence of atom-economical protocols for enantioselective allylations of both aromatic and aliphatic imines, and due to the necessity to pre-synthesise imines. As both challenges in the last decade were addressed to a certain extent, the aza-Cope methodology slipped to the second plan. However, a revival of interest towards the recently developed [1,3]-formal rearrangement approach may be expected as such transformation may offer access to the homoallylic amine scaffolds, difficult to access by the direct allylation methodologies from the two previous classes.

Class (iv) on the other hand, so far is represented by the sole methodology which is capable of producing quaternary stereogenic centres. Despite this methodology is not a classic allylation but rather a Mannich-type reaction, further investigation into the scope and the expansion of this methodology to other substrate classes will be beneficial. Its application to the synthesis of alkaloids and natural products can be also expected.

To draw an endline, it is apparent from the presented work, that metal-free organocatalytic nucleophilic asymmetric allylations of ketimines remains a significant challenge for all four general classes of allylation, the solution of which is likely to be revealed in the forthcoming research.

## Data Availability

Data sharing is not applicable as no new data was generated or analyzed in this study.
